# Giant coronary aneurysms producing chest pain

**DOI:** 10.1186/s13019-019-0872-4

**Published:** 2019-03-08

**Authors:** Raymond Pfister, Yalda Sadeghi, Javier Orrit, René Prêtre

**Affiliations:** Department of Cardiac Surgery, Centre Hospitalier Universitaire Vaudois (CHUV), 1011 Lausanne, Switzerland

**Keywords:** Coronary ectasia, Giant coronary aneurysm, Coronary bypass

## Abstract

**Background:**

Coronary artery aneurysms (CAA) are defined as localized coronary artery dilations more than 1.5 times the diameter of the adjacent segments [1]. Giant coronary aneurysms (GCAA) are unusual and aneurysms on the left side are even rarer. Mechanisms are unclear, but seem predominated by atherosclerosis. Until now, management of giant coronary aneurysm is still unclear.

**Case presentation:**

A 62-year-old man, presented a 4-month history of progressive chest pain aggravated by physical CAAs: 3 on the right coronary artery (RCA), including a giant one, and one on the intermediate branch. Intraoperatively, we found two proximal RCA CAAs of 2 cm each, a 6 cm distal RCA CAA partially thrombosed, and a 3 cm CAA on the intermediate branch.

The two largest CAAs were resected and two saphenous graft bypasses were performed.

**Conclusions:**

Treatment options include medical treatment (antiaggregation, anticoagulation), percutaneous coronary angioplasty and surgery. Results of observational or conservative management in the few cases of GCAA described in literature, appear to have poor results. Surgery is a good option with low operative risk, especially in giant coronary aneurysms.

**Electronic supplementary material:**

The online version of this article (10.1186/s13019-019-0872-4) contains supplementary material, which is available to authorized users.

## Introduction

Coronary artery aneurysm is a rare disease, with an angiographic incidence of 0.15 to 4.9% [1]. CAA is defined as a dilated segment greater than 1.5 times the diameter of an adjacent healthy segment. CAAs are known to be an independent predictor of mortality with an overall 5-year survival of only 71% [2]. Giant CAAs with a diameter greater than 5 cm are exceptions. CAAs are often discovered unexpectedly. Main complications include myocardial ischemia or infarction, rupture being unusual. There is currently no consensus concerning the treatment strategy.

We report the case of a patient with multiple CAA, including a giant one of 6 cm on the RCA and another of 3 cm on an intermediate branch, who successfully underwent resection with coronary bypasses.

## Case report

**Fig. 1:**
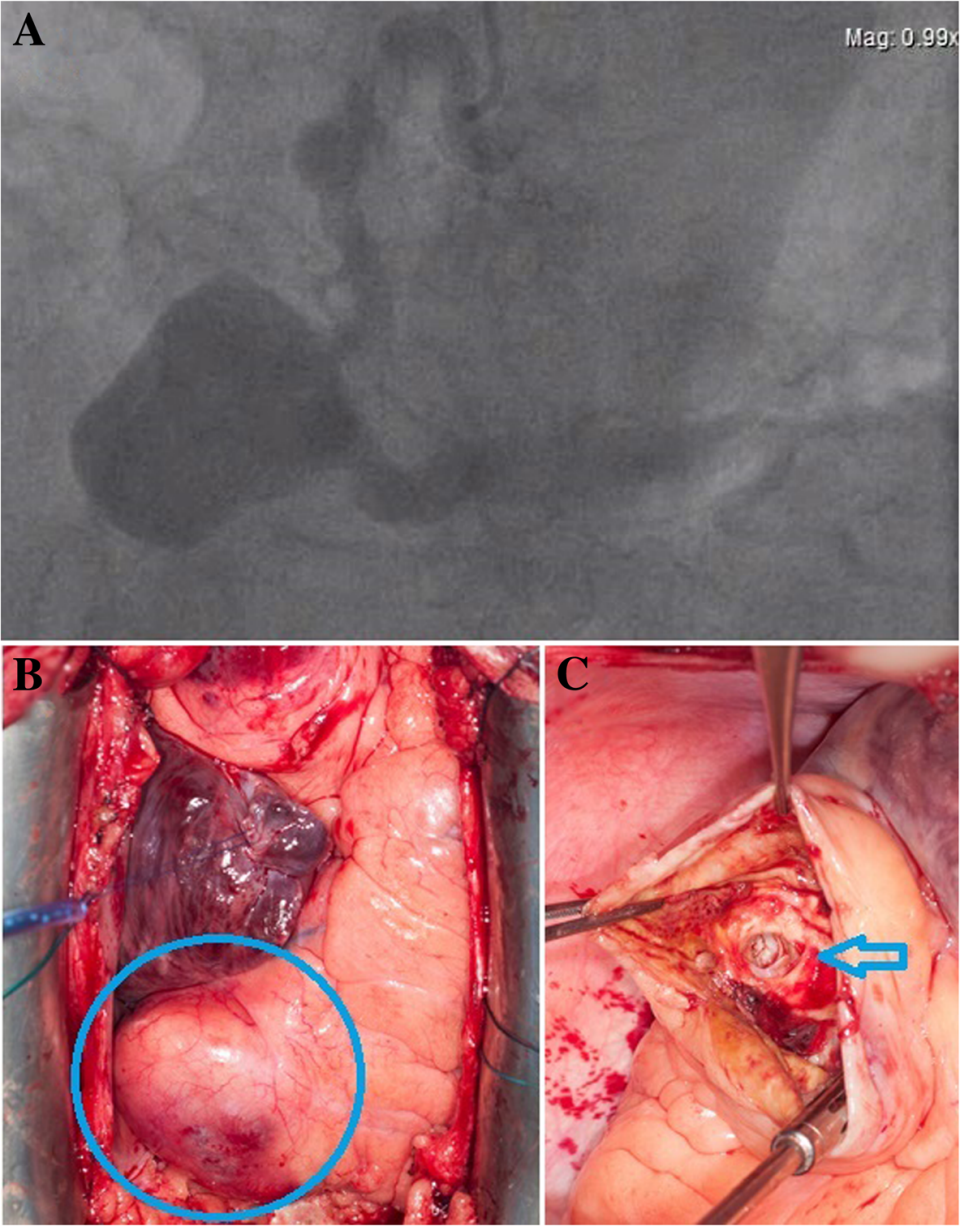
**a** RCA coronarography **b** External aspect of GCAA (Circle) **c** Dilated distal right coronary artery (arrow)

A 62-year old man was referred after the discovery of multiple CAAs. The patient, known for hypertension, hypercholesterolemia, and weight excess, presented, as unique symptom, a 4-months history of progressive chest pain aggravated by physical activity. He had no history of coronary artery or connective tissue disease. There was no history of trauma either. Catheterization showed an atypical coronary artery ectasial disease with the presence of multiple CAAs: the RCA was dilated at three different levels, including the largest aneurism estimated at 6 cm (Fig. 1a,b, Additional file 1: video 1 and Additional file 2: video 2). Another aneurism of 3 cm was localized on the intermediate left coronary branch. Left ventricular ejection fraction was normal. No other arterial disease was found during investigations.


Additional file 1:Video 1. Right coronary angiography, Right Anterior Oblique view. (MOV 57681 kb)



Additional file 2:Video 2. Right coronary angiography, Left Anterior Oblique cranial view. (MOV 72458 kb)


**Fig. 2:**
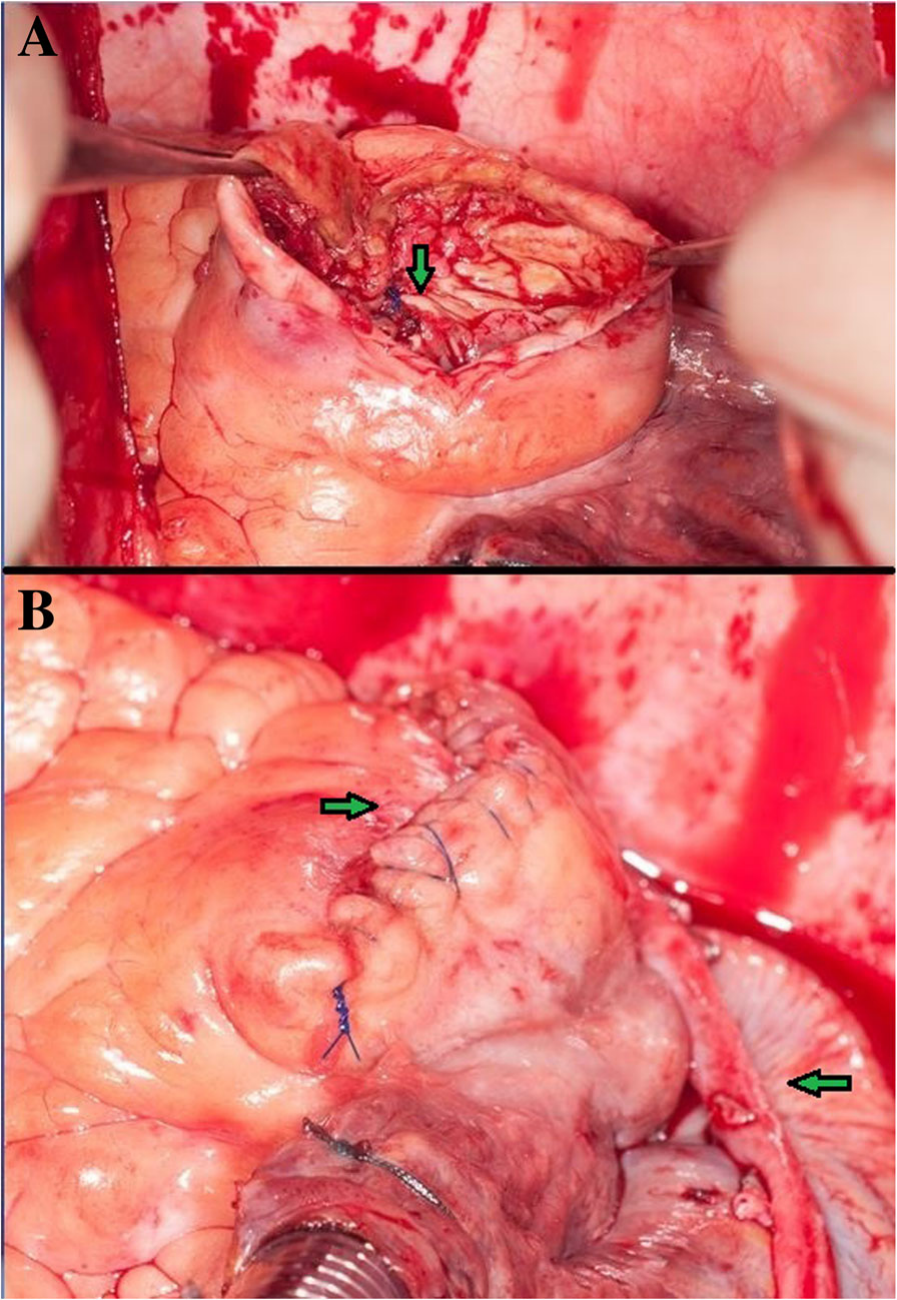
**a** Internal patch closure of the RCA (arrow) **b** Closure of the aneurysm (right arrow) and venous bypass (left arrow)

The operation was performed under central cardio-pulmonary bypass and cardiac arrest by cold crystalloid cardioplegia. Intraoperative, we found two proximal RCA CAAs of 2 cm each, a 6 cm distal RCA GCAA partially thrombosed (Figs. 1 and 2), and a 3 cm CAA on the intermediate branch.

The two largest CAAs were opened, until reaching a normal proximal and distal vessel diameter, resected and inflow ligated. Because of severe calcification the outflow was closed by a pericardial patch, to avoid fragments embolization that ligation could produce. Two saphenous graft bypasses were performed.

## Discussion

Most common cause of CAA is atherosclerosis. Other aetiologies described are congenital, Kawasaki disease, coronary angioplasty, infectious, traumatic and some connective tissue disorders or inflammatory diseases [3, 4]. Keyser et al. suggest that low pressure of the right atrium at the atrioventricular sulcus favorises it on the proximal right coronary artery [3]. In our case, as there is subboclusive coronary artery lesions, GCAA can be related to both inherent defect and poststenotic dilatation [3, 5]. Also, symptoms can be the consequences of either stenosis or embols from the thrombosed GCAA.

Main complications of CAA include thrombosis, embolization and, less commonly, rupture [3]. Independently of coronary artery disease, CAA seems to be itself a risk factor of mortality. Prognosis differs between studies, but most of them show an increase of infarct or mortality connected to CAAs. Baman et al. describes a predicted 5-year mortality of 29.1% and emphasizes the necessity of aggressive approach with coronary risk factors [2]. Therapeutical options for CAAs include medical treatment (antiaggregation or anticoagulation), percutaneous coronary intervention (PCI) and coronary artery bypass (CABG). Although medical treatment seems to have a poorer prognosis, this is not so clear with PCI versus surgery. There is a lack of randomised studies to evaluate outcomes especially on the long term. Concerning isolated CAAs, there is numerous case reports with good short term results with PCI and surgery. But, as it is important to exclude the all aneurysm, covered stents are needed. Again, there is a lack of long term result described, but some late thrombosis are already described [4, 5]. The CASS (Coronary artery surgery study) registry showed similar 5-year survival in patients with CAA and significant coronary stenosis who underwent CABG when compared to patients who had no CAA but underwent CABG for significant coronary stenosis, suggesting a favourable outcome from surgical treatment [4]. No clear evidence exist, but it seems that surgical option should be favoured in cases of bigger (like GCAA), more tortuous and more calcified CAAs.

Based on these results and the anatomic lesions, we opted for surgery. Postoperative course was uneventful. Microbiological analysis was negative and histopathological examination confirmed severe atherosclerosis. 2 years after surgery, the patient get back to work, and didn’t present any symptoms.

## Conclusion

As CAA and GCAAs imply a high mortality rates in conservative management [2, 3] we believe that aggressive management is necessary, by surgery or PCI, depending of the of the global coronary disease of the patient and the complexity of the lesions, with more complexity favoring surgery.

## Abbreviations

CAA: Coronary artery aneurysms
CABG: Coronary artery bypass
CASS registry: Coronary artery surgery study registry
PCI: Percutaneous coronary intervention
RCA: Right coronary artery
